# Characterization of the Growth of *Chlamydia trachomatis* in *In Vitro*-Generated Stratified Epithelium

**DOI:** 10.3389/fcimb.2017.00438

**Published:** 2017-10-10

**Authors:** Ana T. Nogueira, Kristin M. Braun, Rey A. Carabeo

**Affiliations:** ^1^Program in Microbiology, Institute of Medical Sciences, University of Aberdeen, Aberdeen, United Kingdom; ^2^School of Molecular Biosciences, College of Veterinary Medicine, Washington State University, Pullman, WA, United States; ^3^Centre for Cutaneous Research, Blizard Institute, Barts and the London School of Medicine and Dentistry, London, United Kingdom

**Keywords:** *Chlamydia*, organotypic cultures, 3D culture, squamous stratified epithelium

## Abstract

*Chlamydia* infection targets the mucosal epithelium, where squamous and columnar epithelia can be found. Research on *Chlamydia*-epithelia interaction has predominantly focused on columnar epithelia, with very little known on how *Chlamydia* interacts with the squamous epithelium. The stratification and differentiation processes found in the squamous epithelium might influence chlamydial growth and infection dissemination. For this reason, three-dimensional (3D) organotypic stratified squamous epithelial cultures were adapted to mimic the stratified squamous epithelium and chlamydial infection was characterized. *Chlamydia trachomatis* infection in monolayers and 3D cultures were monitored by immunofluorescence and transmission electron microscopy to evaluate inclusion growth and chlamydial interconversion between elementary and reticulate body. We observed that the stratified epithelium varied in susceptibility to *C. trachomatis* serovars L2 and D infection. The undifferentiated basal cells were susceptible to infection by both serovars, while the terminally differentiated upper layers were resistant. The differentiating suprabasal cells exhibited different susceptibilities to serovars L2 and D, with the latter unable to establish a successful infection in this layer. Mature elementary body-containing inclusions were much more prevalent in these permissive basal layers, while the uppermost differentiated layers consistently harbored very few reticulate bodies with no elementary bodies, indicative of severely limited bacterial replication and development. For serovar D, the differentiation state of the host cell was a determining factor, as calcium-induced differentiation of cells in a monolayer negatively affected growth of this serovar, in contrast to serovar L2. The apparent completion of the developmental cycle in the basal layers of the 3D cultures correlated with the greater degree of dissemination within and the level of disruption of the stratified epithelium. Our studies indicate that the squamous epithelium is a suboptimal environment for growth, and thus potentially contributing to the protection of the lower genital tract from infection. The relatively more fastidious serovar D exhibited more limited growth than the faster-growing and more invasive L2 strain. However, if given access to the more hospitable basal cell layer, both strains were able to produce mature inclusions, replicate, and complete their developmental cycle.

## Introduction

Three-dimensional (3D) organotypic stratified squamous models (3D cultures) have been used for decades in the field of epithelial biology to model the stratified squamous epithelium found in the human skin (Bell et al., 1981; Fusenig et al., 1983; Smola et al., 1993; Stark et al., 1999). This tissue is characterized by multiple layers of cells where the proliferative and renewing basal layer divides asymmetrically to form an upper layer of non-replicating suprabasal cells that begin to undergo differentiation (Fuchs and Raghavan, 2002; Simpson et al., 2011). During the transit between the basal layer to the surface, morphologic, and biochemical changes occur in the keratinocyte cells including the reduced expression of keratin 14 (K14) and the activation of specific differentiation markers, including keratin 10 (K10), filaggrin, involucrin, and others (Fuchs and Green, 1980; Steinert et al., 1981; Moll et al., 1982, 2008; Eckert and Rorke, 1989; Fuchs, 1990; Sandilands et al., 2009; Eckhart et al., 2013). More recently stratified squamous epithelium have been applied in microbiology to investigate bacterial, fungal, and viral species interactions with the skin (Schaller et al., 2000; Groeber et al., 2011; de Breij et al., 2012; Soong et al., 2012; Hogk et al., 2013; Popov et al., 2014). Although, keratinocytes from the skin and the keratinocytes from the mucosae go through the differentiation process in a similar way, mucosal keratinocytes neither keratinize nor enucleate at the final stage of differentiation (Kikuchi et al., 1997; Patton et al., 2000; Anderson et al., 2014). For this reason, in contrast to skin tissues, the uppermost layers in mucosae epithelium do not contain a layer of dead cells. Instead, it has the characteristics of mucosal tissues of the lower genital tract, oral cavity, esophagus, corneal epithelium, rectum, and foreskin (Chateau and Boehm, 1996; Kikuchi et al., 1997; Anderson et al., 2014).

*Chlamydia trachomatis (C. trachomatis)* is one of the most common bacterial sexually transmitted infections (Kreisel et al., 2017). The World Health Organization estimates over 100 million *C. trachomatis* infections worldwide (WHO, 2014). *Chlamydia* is an obligate intracellular pathogen that is characterized by a unique biphasic developmental cycle. The elementary body (EB), which is the extracellular form of *Chlamydia*, has the ability to attach and invade susceptible cells. Once inside the host cell and within a membrane-bound vacuole called an inclusion, EBs differentiate into reticulate bodies (RBs), the metabolically active and replicative, but non-infectious form. After several cycles of replication RBs begin to differentiate back into EBs, which are subsequently released to infect neighboring cells (AbdelRahman and Belland, 2005).

The primary site of infection of *C. trachomatis* is the epithelial mucosae. This bacterium targets epithelia that are often composed of a single layer of columnar cells or those found in the transformation zone in proximity with a stratified non-keratinising squamous epithelium, which includes the genital tract, the anorectum, and nongenital sites. It is better understood how *Chlamydia* species establish infection and disease in the columnar epithelium, but chlamydial interaction with the non-keratinising squamous epithelium has not been investigated to the same degree. Studies on the host interaction of *C. trachomatis* at the cellular level have been primarily carried out in monolayer cultures of epithelial cells due to the majority of clinical studies in women reporting the predominance of active infection to the upper genital tract (Taylor and Haggerty, 2011). *Chlamydia* infection in the lower genital tract is relatively absent compared to the upper genital tract, despite the lower genital tract environment being anti-inflammatory in character (Lee et al., 2015). This indicated that the relative resistance of the lower genital tract would involve factors unrelated to the host immune response. Indeed, previous work by Moorman et al. suggested the existence of these factors by investigating chlamydial growth properties from disaggregated endometrial and ectocervical tissues and reporting the existence of less efficient growth in squamous epithelial cells relative to those columnar epithelial cells obtained from the endocervix. Additionally, increased susceptibility to *Chlamydia* infection is linked to ectopy, where a portion of the endocervix, which consists of a single layer of columnar epithelial cells, becomes exposed in the ectocervix (Lee et al., 2006). These observations hint at the chlamydial preference for columnar over stratified squamous epithelium. From these historical evidence, we hypothesize that the lower genital tract functions as a barrier, whose function is related to the stratified and possibly differentiating character of the resident squamous epithelium.

To investigate this hypothesis, we employed the spontaneously immortalized, but non-transformed keratinocyte epithelial cell line, HaCaT. These cells retain relevant differentiation properties to differentiate, either through exposure to high calcium (Ca^2+^) in the growth media or by seeding on collagen gels with embedded fibroblasts, and raising the confluent monolayer to the liquid-air interface. The exposure to the liquid-air interface simultaneously induces stratification and differentiation, forming an organized three-dimensional culture with well-defined layers at various states of differentiation that resemble non-keratinizing squamous epithelium from mucosae (Boukamp et al., 1988; Boelsma et al., 1999; Stark et al., 1999, 2004). This characteristic of HaCaT cells has been invaluable in cell biology and in pathogenesis studies of the Human Simplex virus (HSV). In contrast to HaCaT cells, other human epithelial cell lines transformed by viral oncogenes affect their differentiation and ability to stratify properly (Blanton et al., 1991; Tsunenaga et al., 1994). Moreover, HaCaT cells when seeded as monolayers, were able to support chlamydial growth (Joubert and Sturm, 2011). Hence, for studies that interrogate the effects of the stratified architecture and cell differentiation on *Chlamydia trachomatis* infection, HaCaT cells are the most appropriate.

In this report, we used a three-dimensional (3D) stratified epithelial model to gain insight into the chlamydial biology during infection of squamous epithelia. We considered the stratification and states of differentiation of HaCaT cells in chlamydial growth and replication. During infection of 3D cultures by *C. trachomatis*, we observed that the degree to which chlamydial development progressed depended highly on which layers of cells within the 3D cultures were infected. In other words, the different layers of the *in vitro*-generated stratified epithelium displayed different levels of susceptibility in the detection of a complete chlamydial developmental cycle. Infection of basal cells with *C. trachomatis* consistently yielded inclusions containing EB-like bodies, which disseminated throughout the stratified epithelium in a penicillin-sensitive manner. Dissemination significantly disrupted the stratified organization of the raft culture. In contrast, while *C. trachomatis* were able to infect the uppermost layers consisting of terminally differentiated cells, their development was significantly delayed. However, chlamydial organism numbers were markedly reduced, and no differentiation to infectious EBs was observed up to 5 days of infection. Thus, the interaction of *C. trachomatis* with stratified epithelia may be more complex, and likely involves factors related to the terminal differentiation state of the host cell and/or the stratified configuration of the epithelium.

## Materials and methods

### Cell culture

HeLa and NIH 3T3 fibroblast cell lines were cultured in Dulbecco's Modified Eagle Medium (DMEM) (11960-044, Gibco by Life technologies) supplemented with 10% fetal bovine serum (F0926, Sigma), 20 mM L-glutamine (25030081, Gibco by Life technologies) and 10 μg/mL gentamicin (Life technologies). For subcultures, cells were trypsinised with 0.05% trypsin-EDTA solution (Life technologies). HaCaT cells were cultivated in 1 part F12 (11765-054, Gibco by Life technologies) and 3 parts DMEM supplemented with 10% fetal bovine serum FCS-SA, Labtech, 10 μg/mL gentamicin (Life technologies), 20 mM L-glutamine, Insulin (I9278, Sigma), and HCE cocktail containing hydrocortisone (AC35245-0010, Fisher Scientific), cholera toxin (C8052, Sigma) and epidermal growth factor (AF-100-15, PeproTech) (final concentrations in media were 10^−10^ M, 0.5 μg/mL and 10 ng/mL, respectively; Celis, 2006). Cells were trypsinised with 0.5% trypsin-EDTA (10x) solution (15400-054, Gibco by Life technologies) diluted to 5x with versene solution (15040-066, Gibco by Life technologies). All cell lines were cultured at 37°C with 5% CO_2_. To induce HaCaT differentiation, cells were grown until confluence, and cell culture media was substituted by DMEM:F12 (Life technologies) supplemented with 1% FBS and 2 mM calcium chloride (CaCl_2;_ Sigma).

### Chlamydial propagation and infections

HeLa cells were used to propagate *C. trachomatis* serovar L2 (LGV-434) (*C. trachomatis* L2) or *C. trachomatis* serovar D (*C. trachomatis* D) (kindly provided by Dr. Grieshaber, University of Idaho). Infected cells were harvested, and *C. trachomatis* was purified by discontinuous density gradient centrifugation in gastrografin (Bayer). For infection, cell culture monolayers were plated to 70–80% confluence on a glass coverslip except for the calcium-differentiated experiments where HaCaT cells were plated to 100% confluence. Monolayers were infected at an MOI (multiplicity of infection) of 1 or 5 and centrifuged for 5 min at 1,000 rpm, 4°C, unless otherwise stated. Negative controls (mock-infected) were incubated with cell culture media only. Cells were incubated at 37°C with 5% CO_2_ for the specific time points. Inoculum for 3D cultures infection was calculated considering a confluent monolayer, and were infected at an MOI of 5 by introducing *C. trachomatis* inoculum (10^9^ IFU/mL for C. trachomatis L2 and 10^8^ IFU/mL *C. trachomatis* D) at the top of the cultures in a total volume of 80 μL per culture. The cultures were allowed to rest for 15 min prior to returning them to the incubator for the specific time points. For penicillin experiments, 3D cultures were infected at the day of liquid-air interface. After 2 h of infection, media containing penicillin (final concentration 100 μL/mL, ~15 U/mL) was added to the bottom of the insert. For each time point of penicillin recovery, wells were washed with cell culture media twice followed by the addition of fresh cell culture media until the end of the experiment.

The experiments were conducted following the appropriate protocols commensurate with Biosafety Level 2 procedures.

### Quantification of recoverable infectious particles

Triplicates of infected monolayers were washed with Hank's Balanced Salt Solution (HBSS) (Gibco by Life technologies). Cells were lysed by addition of ice-cold water. Cell suspensions were diluted (1:50) in the adequate cell culture media and used to re-infect fresh monolayers, in triplicate. Plates were incubated for 1 h at 37°C with 5% CO_2_ and the inoculum replaced with fresh cell culture media. Plates were returned to the incubator for an additional 24 h. Samples were then fixed with 4% PFA (Sigma) for 15 min at room temperature (RT) and washed three times with Phosphate Buffered Saline (PBS).

### Three-dimensional (3D) organotypic stratified squamous cultures

A collagen solution was prepared following the manufacturers protocol for RAFT™ reagent kit (016-0R94, Lonza) for 24-well plates. When indicated, NIH 3T3 fibroblasts were added to the collagen solution and pipetted to a 24-well culture plate. Plates were incubated at 37°C for 15 min. After incubation, absorbers were applied on top of each collagen gel for 15 min. Then, the absorbers were removed and cell culture medium containing 3 × 10^5^ cells/mL of HaCaT cells were added. Plates were incubated at 37°C with 5% CO_2_ in submerged cell culture media until cells reached confluence. Cell-seeded collagen gels were transferred to 6-well plate cell culture inserts (PICM03050, Millicell®) at the air-liquid interface (non-submerged) for the indicated times. Media was changed every other day. On the specified time points, the cell culture wells were washed three times with HBSS and OCT-embedded (23-730-571, Fisher Scientific) followed by rapid freeze in isopentane chilled with liquid nitrogen. There were variability in the raft culture thickness, with the majority of the observed differences ranging from 1 to 2 cell layers, but minimal variability in differentiation. One possible cause is how many times the HaCaT cells were passaged. We did not use HaCaT cells passaged beyond 15 passages.

### Haematoxylin and eosin (H&E) staining and immunofluorescence staining

Cryo-embedded sections were washed with distillated water for 5 min. Nuclei were stained with haematoxylin for 5 min, washed in tap water, differentiated by immersing in 1% acid alcohol, and rinsed in water. The cytoplasm was stained with eosin for 5 min. Sections were dehydrated by ascending grades of ethanol followed by xylene. Sections were mounted with Immu-Mount mounting medium (Thermo Scientific). Brightfield pictures were obtained on the Leica Camera w/Leitz & Fluorescence Stereo Microscope. Immunofluorescence was performed on frozen and OCT embedded sections. Sections were air-dried and then fixed with 4% PFA for 15 min. Cells grown on glass coverslips were rinsed three times with PBS and fixed with 4% PFA for 15 min at RT. Fixed samples were rinsed with PBS and permeabilized with 0.2% Triton X-100 in PBS for 5 min at RT with agitation. Samples were washed three times with PBS, and blocked with 5% BSA for 30 min, at RT. Then, sections or coverslips were incubated with anti-K14 (ab51054, Abcam), anti-K10 (ab76318, Abcam), anti-*Chlamydia* LPS (ab21180 or ab62708 from Abcam or NB100-62449 from Novus), anti-*Chlamydia* (PA1-25652 from Thermo Fisher Scientific) anti-filaggrin (ab3137, Abcam) or (ab92742, Abcam). Phalloidin (A12379, Thermo Scientific) and DAPI were used to stain F-actin and DNA, respectively. Samples were then rinsed 3 times with PBS and incubated with appropriate Alexa Fluor conjugated secondary antibodies (Thermo Fisher Scientific). Coverslips and sections were mounted onto microscope glass slides using Immu-Mount mounting medium (Thermo Scientific). All samples visualization was performed on a laser scanning confocal microscope (Leica SP8 Point Scanning Confocal or Leica SP8-X White Light Point Scanning Confocal). Images were processed using ImageJ.

### Transmission electron microscopy

3D cultures were washed twice with HBSS. Cultures were fixed with 2.5% glutaraldehyde in Phosphate Buffered Saline (PBS) for 1 h at 4°C. Fixed cultures were washed 3 times with PBS for 5 min each and post-fixed with 1% osmium tetroxide in PBS for 30 min. Then, samples were washed 3 times with distilled water for 10 min to remove the excess of phosphate. Cultures were dehydrated in graded ethanol starting with 50, 70, 95, and 100% for 30 min each followed by infiltration with ethanol/Spurr's (1:1) solution. After 1 h, a fresh infiltration solution of ethanol/Spurr's (1:10) was added for an additional 60 min. 3D cultures were embedded in fresh Spurr's resin, placed in a 60°C oven, and polymerized overnight. Seventy to ninety nanometers of thick sections were cut on Leica UC6 or Reichert ultramicrotome. Samples were visualized on a JEOL 1400 plus + AMT UltraVUE camera and FEI TEM T20.

### Western blots

HaCaT cells were collected at the time of differentiation induction as a time-point zero, and then at 2, 3, 5, or 7 days post-differentiation. For each time-point, cells were washed in PBS and then lysed with RIPA buffer (AAJ60780EQE, Fisher Scientific) supplemented with protease inhibitor (Roche Diagnostics) and phosphatase inhibitor (Roche Diagnostics), following manufacturer's instructions. Samples were incubated at 4°C for 30 min followed by centrifugation at 14,000 × g for 20 min at 4°C. After supernatants were collected, protein concentrations were determined using Bradford's assay. Samples were diluted in 5x SDS sample buffer and boiled for 10 min at 100°C. Samples were resolved in a SDS 10% polyacrylamide gels and transferred to a nitrocellulose membrane (Whatman) for 60 min at 100 V by the standard wet transfer method. Immunoblotting was performed using primary antibodies against cytokeratin 10 (K10) and cytokeratin 14 (K14) (ab76318 and ab51054, respectively from Abcam), followed by the secondary antibodies polyclonal goat anti-rabbit and polyclonal rabbit anti-mouse conjugated to HRP (DAKO). Tubulin antibody (ab21058, Abcam) was used as a loading control.

## Results

### 3D culture establishment and regular expression of differentiation markers

HaCaT cells were plated onto the surface of collagen gels containing embedded NIH 3T3 fibroblasts, and cultured at the liquid-air interface. Previous studies showed that NIH 3T3 fibroblasts employed as feeder cells supported tissue architecture and terminal differentiation in a similar way as human dermal fibroblasts (Smola et al., 1993; Maas-Szabowski et al., 2001). Different concentrations of fibroblasts were tested to determine the optimum number required to achieve multi-layered and differentiated epithelia. Cultures with 0, 2 × 10^4^, or 4 × 10^4^ fibroblasts embedded in collagen were grown and differentiated in air-exposed cultures for 14 days (Supplementary Figure 1). Raft cultures were fixed and processed for histochemical staining. Samples cultured in the absence of fibroblasts exhibited less stratification as compared to those co-cultured with higher number of fibroblasts (Supplementary Figure 1). We also determined that in our hands, full stratification, which was defined as the formation of several layers of squamous cell in the uppermost region at 14 days of culture, required at least 4 × 10^4^ fibroblasts embedded in the 100-μm collagen matrix (Supplementary Figures 1, 2). Enucleated cells were not common, and keratinization was incomplete. These are typical features of non-keratinizing epithelia (Chateau and Boehm, 1996; Kikuchi et al., 1997; Anderson et al., 2014).

To investigate the differentiation of HaCaT cells when raised to the air-liquid interface, the expression of specific keratins was examined. Cryo-embedded sections were analyzed by immunofluorescence for specific differentiation markers, including keratin 14 (K14), keratin 10 (K10), and filaggrin. We also monitored the expression of the nuclear protein ki67, which indicated cell proliferation (Supplementary Figure 2). Cells in the basal and suprabasal layers were positive for K14. Ki67 signal was predominantly associated with the basal layer. K10 staining demonstrated some overlap with that of K14, indicating cells transitioning between differentiation states. As expected, ki67 signal did not overlap with K10. Filaggrin could be observed in the upper layers, with partial overlap with K10. We also observed well-formed desmosomes by transmission electron microscopy (Supplementary Figure 2). In summary, our 3D cultures showed the following markers for the different layers: ki67, K14, K10, and filaggrin, with some layers exhibiting overlaps of each marker tested. Together with the well-formed desmosomes, these observations indicate that our *in vitro*-generated stratified epithelia mimic important differentiation and stratification features of squamous epithelia.

### Growth of *C. trachomatis* in human keratinocytes

Previous work showed that HaCaT cells were able to support chlamydial growth (Joubert and Sturm, 2011). We confirmed this finding by analyzing the production of inclusion forming units (IFU), and inclusion morphology. Both HaCaT and HeLa cells were infected with *C. trachomatis* L2 at an MOI of 1. Inclusion development was monitored at 2, 20, and 36 h post-infection (h p.i.), and visualized by indirect immunofluorescence using anti-chlamydial LPS antibody, followed by confocal microscopy (Supplementary Figure 3A). Inclusions within HeLa and HaCaT cells were very similar in appearance. We then determined if both cell lines could equally support the completion of the developmental cycle, i.e., production of infectious particles (EBs) (Supplementary Figure 3B). Infectious particles were harvested at different time points by hypotonic lysis, and the recovered infectious particles were added to a fresh monolayer of HeLa cells to determine harvest titers. Infection was allowed to proceed for 24 h, and samples were processed for immunofluorescence to visualize and enumerate inclusions. Both HeLa and HaCaT cells produced similar inclusion forming units (IFU) (10^7^ IFU/mL) at the latest time point monitored (36 h p.i.; Supplementary Figure 3B). The results indicated that in a monolayer configuration, HaCaT cells equally supported chlamydial development and replication as HeLa cells.

### *Chlamydia trachomatis* infection is delayed in fully stratified cultures

**Figure 1:**
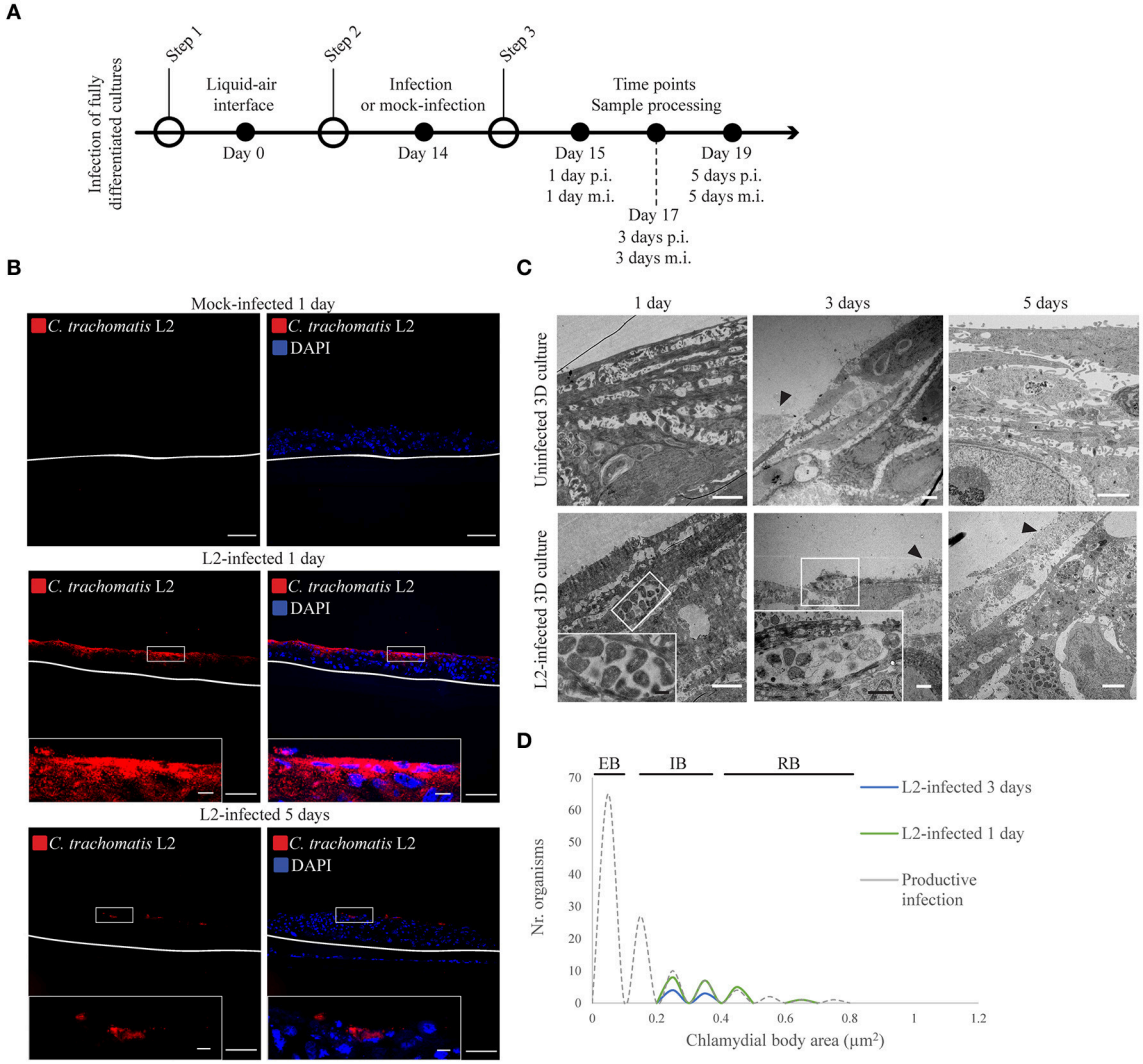
*Chlamydia trachomatis* infection is delayed in 3D organotypic cultures. **(A)** Diagram of the 3D cultures set up for the differentiation, stratification, and infection stages. **(B)** *C. trachomatis* L2 inclusions (red) in organotypic cultures infected for 1 or 5 days were only present on the top-most layers. An anti-*C. trachomatis* LPS antibody was used to detect inclusions. White line represents the bottom of the 3D culture. A representative image from four independent experiments is shown. Scale bar = 10 μm. **(C)** TEM micrographs show *C. trachomatis* L2 inclusions containing only normal-size RBs independently of the time of infection, 1, 3, or 5 days. Black arrowheads point at cells that were either being shed or undergoing cell death. A representative image from three independent experiments is shown. **(D)** Quantification of inclusion contents and the area of elementary bodies (EBs), reticulate bodies (RBs) and intermediate bodies (IB) in 3D cultures infected from the top for 1 (green line) or 3 days (blue line). The dotted line indicates the area distribution for chlamydial organisms in productive infections. White scale bar = 2 μm; black scale bar in inset = 500 nm.

The stratified squamous tissue has a more complex epithelial organization given its various stratification and differentiation states. Three-dimensional organotypic stratified epithelial cultures (3D cultures) of HaCaT were infected, and *C. trachomatis* growth assessed. Air-exposed cultures were allowed to stratify and differentiate for 14 days prior to infection. On the 14th day, the inoculum was directly applied to the top of the stratified epithelia where terminally differentiated cells were located (Supplementary Figure 2). The inoculum was calculated by considering a confluent monolayer at the top of the 3D cultures, and 80 μL was introduced on top of each 3D culture. Infection was allowed to proceed for 1 or 5 days for *C. trachomatis* L2 (Figure 1A) and 1 or 3 days for *C. trachomatis* D (Supplementary Figure 4) before sample processing and analysis. Cryosections were stained for *C. trachomatis* using an anti-*Chlamydia* LPS antibody. *C. trachomatis* L2 inclusions were only observed in the top layers of the stratified epithelia with no apparent dissemination to the lower layers for both 1 and 5 days post-infection (d p.i.) samples (Figure 1B). Similarly, *C. trachomatis* D infection showed no apparent dissemination, and in contrast to *C. trachomatis* L2, inclusions were almost absent by immunofluorescent analysis by 1 d p.i. (Supplementary Figure 4). By 3 d p.i. no inclusions were detected for *C. trachomatis* D, which precluded analysis of developmental forms by transmission electron microscopy (TEM). We evaluated the morphology of *C. trachomatis* L2 developmental forms at 1, 3, and 5 d p.i. (Figure 1C) from three independent experiments. The majority of the inclusions contained small numbers of reticulate bodies (RBs) in both 1 and 3 days post-infection samples. We also observed very few infected cells in the top layer in the 3 day samples, which could be attributed to the natural shedding occurring in the uppermost layer of the epithelium (Figure 1C, black arrowheads). By 5 days post-infection no inclusions were found by TEM. We examined the morphology of *C. trachomatis* L2 forms within the inclusions, and quantified the area for each form. We measured the area rather than the diameter given the irregular shape of some of the RBs. RBs that appeared to be undergoing binary fission were excluded. TEM images across three independent experiments were obtained for 1 and 3 d p.i. when possible. Previous studies have established that the area of an RB is between 0.4 and 0.8 μm^2^, and the area of an EB is expected to be between 0.07 and 0.12 μm^2^ (Wyrick, 2010). Indeed, from cells containing newly formed EBs, such as those shown in **Figure 3**, we found our results to be consistent with previous reports (Figure 1D). The inclusions found in the uppermost layers of the 3D epithelium contained Chlamydiae with areas between 0.25 and 0.65 μm^2^ (Figure 1D), which are consistent with the area of an intermediate body (IB), although we could not exclude the possibility that some of the organisms categorized as IBs may instead be RBs sectioned above or below the “equator.” Overall, when compared against the growth kinetics obtained for HeLa and HaCaT cells in the monolayer configuration, *C. trachomatis* development was severely delayed in the stratified and differentiated HaCaT cells. Despite allowing for the infection to progress for up to 5 days, the inclusions remained morphologically immature, with very small numbers of Chlamydiae residing within the cells, and the absence of late developmental forms, e.g., elementary bodies. This was in contrast to inclusions in HaCaT monolayers, where the majority was readily visible with multiple organisms after 1 day post-infection (Supplementary Figure 3).

### *Chlamydia trachomatis* infection in calcium-differentiated HaCaT

**Figure 2:**
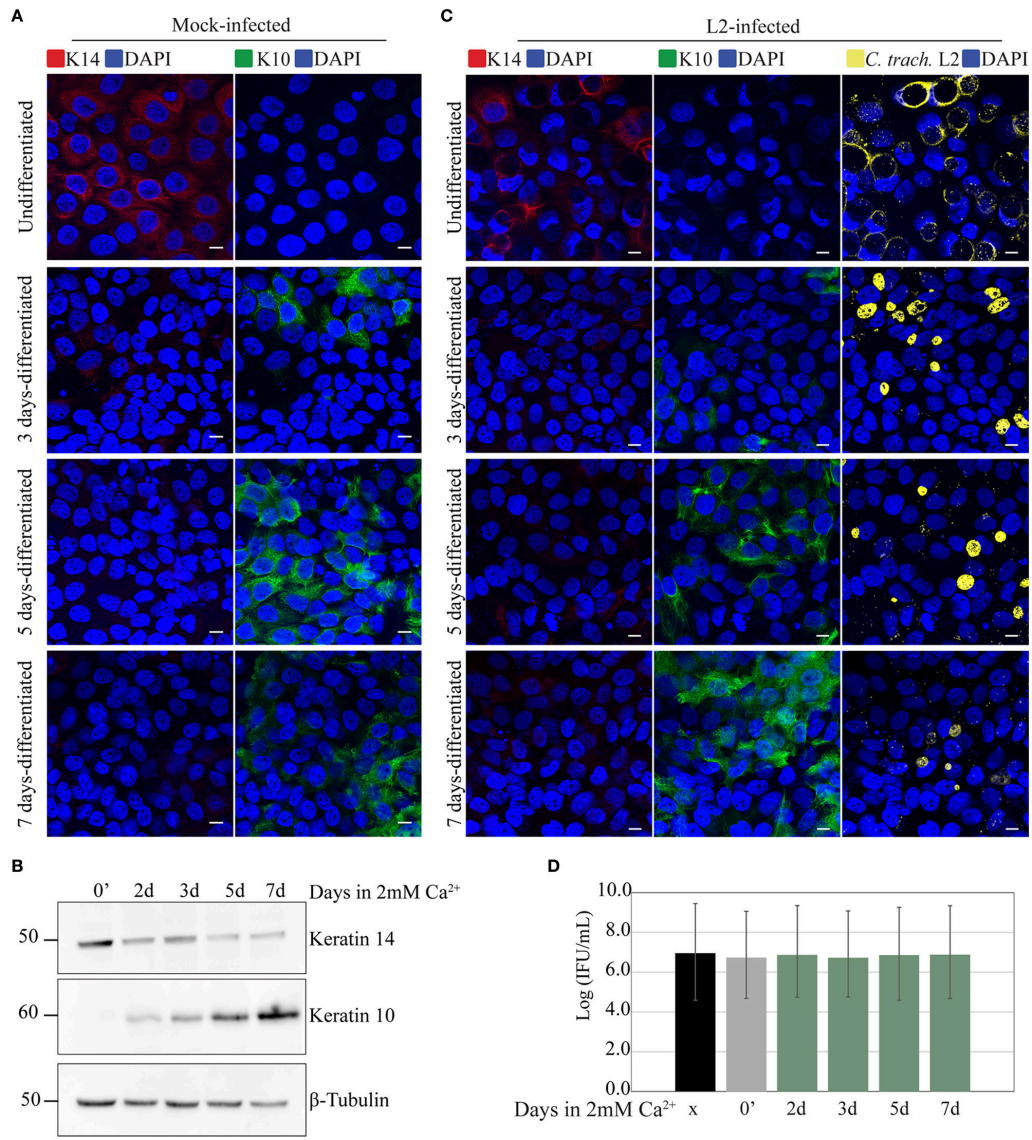
Calcium-induced differentiation of HaCaT cells does not affect *C. trachomatis* growth and development. HaCaT cells in a monolayer system were exposed to 2 mM of calcium (Ca^2+^) to induce differentiation up to 7 days. **(A)** The marker K14 is highly present in undifferentiated cells, and decreased after calcium exposure. In contrast K10 expression increased after calcium exposure indicating differentiation. All images were subjected to identical processing parameters using NIH ImageJ. A representative image from three independent experiments is shown. Scale bar = 10 μm. **(B)** Differentiation with calcium exposure was confirmed by western blot. Keratin 14 expression is decreased, while keratin 10 expression is increased with 2 mM Ca^2+^ exposure. β-tubulin served as the loading control. A representative image from two independent experiments is shown. **(C)** Undifferentiated HaCaT (0′ calcium exposure) or HaCaT exposed to calcium were infected with *C. trachomatis* L2 for 24 h without centrifugation-synchronized step. Chlamydial inclusions in all conditions appeared mature. *C. trachomatis* inclusions were stained with covalescent human sera (in yellow). A representative image from two independent experiments is shown. Scale bar = 10 μm. **(D)** Inclusion forming unit (IFU) yields are similar for untreated and Ca^2+^-treated samples, a measure for chlamydial infectious particles. HeLa, undifferentiated HaCaT (0′ calcium exposure) or HaCaT pre-exposed to calcium at the indicated times were infected for 36 h, and IFU yield enumerated. Data is presented as mean IFU ± *SD*.

It is possible that the observed delay in chlamydial growth in stratified epithelia was due to the differentiation state of the cells. To investigate this, we took advantage of a known property of HaCaT cells—the induction of differentiation by the addition of high calcium concentration to the culture medium (Hennings et al., 1980; Deyrieux and Wilson, 2007). After seeding on coverslips and growing to full confluence, media containing 1% FBS supplemented with 2 mM Ca^2+^ was added to the cells to induce differentiation. Differentiation was confirmed independently by monitoring the changes in expression of the keratinocyte differentiation markers K10 and K14 by fluorescence microscopy and Western blot. In samples exposed to high [Ca^2+^], we observed decreased expression of K14 along with the concomitant increase in K10 expression. In the untreated controls, cells maintained the expression of K14, and remained negative for K10 (Figures 2A,B). In a parallel experiment, Ca^2+^-differentiated HaCaT cells were infected with *C. trachomatis* serovar L2 by inoculating the monolayer without centrifugation (Figure 2C). *C. trachomatis* L2 were introduced to cultures and incubated for 1 h at 37°C followed by a washing step to removed unadhered EBs. Fresh media was added, and the cells incubated for 24 h. As shown in Figure 2C, serovar L2 was able to infect and form inclusions regardless of the duration of differentiation. Inclusion sizes were heterogeneous, which is likely due to the asynchronous method of infection, e.g., without centrifugation. To better assess chlamydial development, we synchronously inoculated with serovar L2 samples of HaCaT monolayers at different times of Ca^2+^-induced differentiation, including an undifferentiated control, and monitored IFU production at 24 h p.i. HeLa cells were used as control. All treatment groups yielded similar levels of IFU (Figure 2D). For this reason, we concluded that Ca^2+^-induced differentiation of HaCaT cells had no effect on *C. trachomatis* serovar L2 growth and development.

### Incompletely stratified cultures were productively infected with *Chlamydia trachomatis* serovar L2

**Figure 3:**
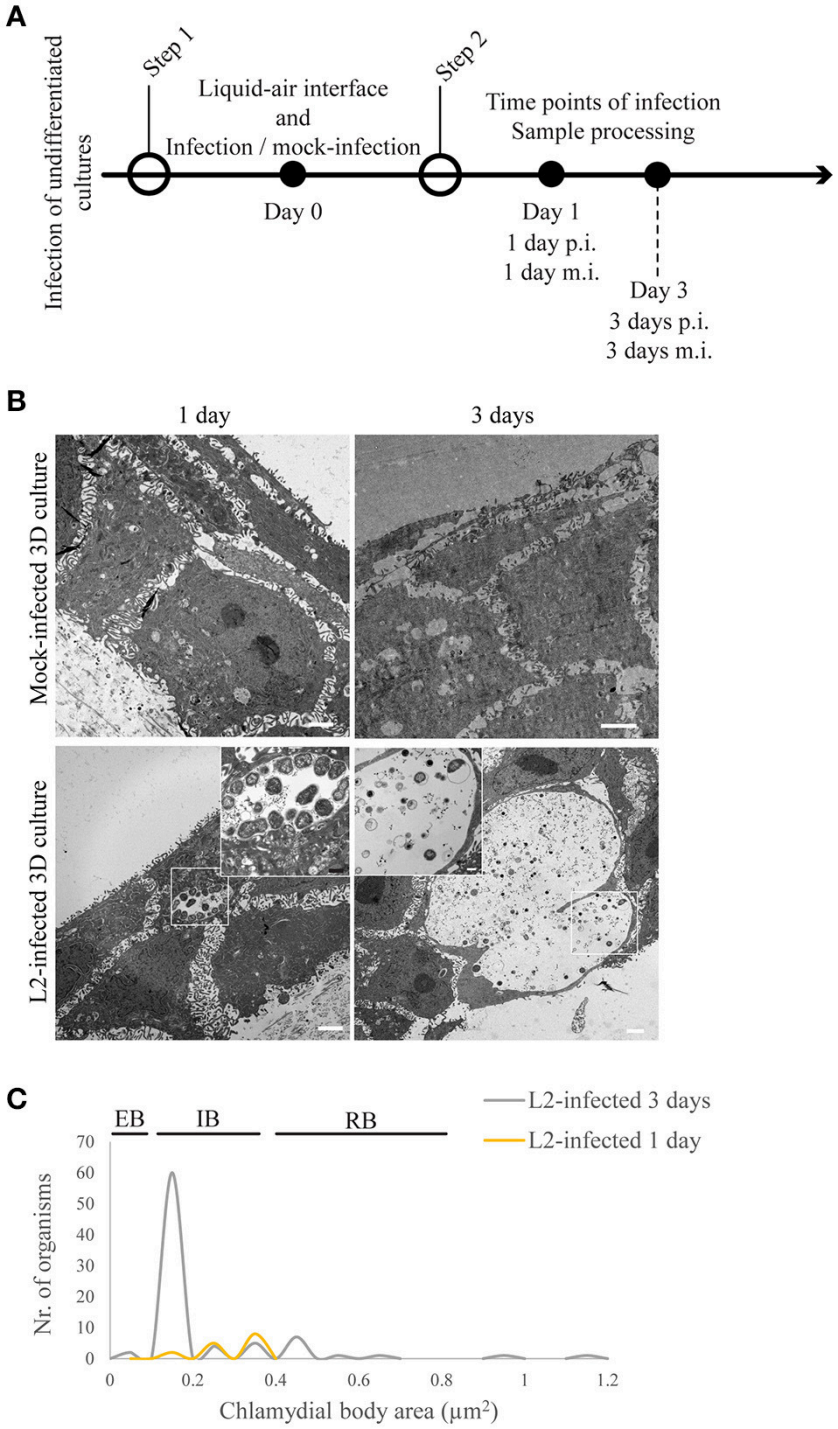
*Chlamydia trachomatis* completes its developmental cycle in undifferentiated layer of the 3D organotypic cultures. **(A)** Diagram of the 3D cultures differentiation, stratification and infection stages. **(B)** Undifferentiated/basal layer was infected with *C. trachomatis* L2 prior to differentiation to mimic basal layer access through microabrasions. TEM micrographs show inclusions containing normal-size RBs 1 day post-infection. By 3 days of infection inclusions contains EBs. Cultures allowed to stratify for 19 days in the presence of *C. trachomatis* L2 revealed few layers of unorganized cells. A representative image from two independent experiments is shown. **(C)** Quantification of inclusion contents and the area of EBs, RBs, intermediate bodies and enlarged RBs. The plot indicates the number of organisms for each particle size.

**Figure 4:**
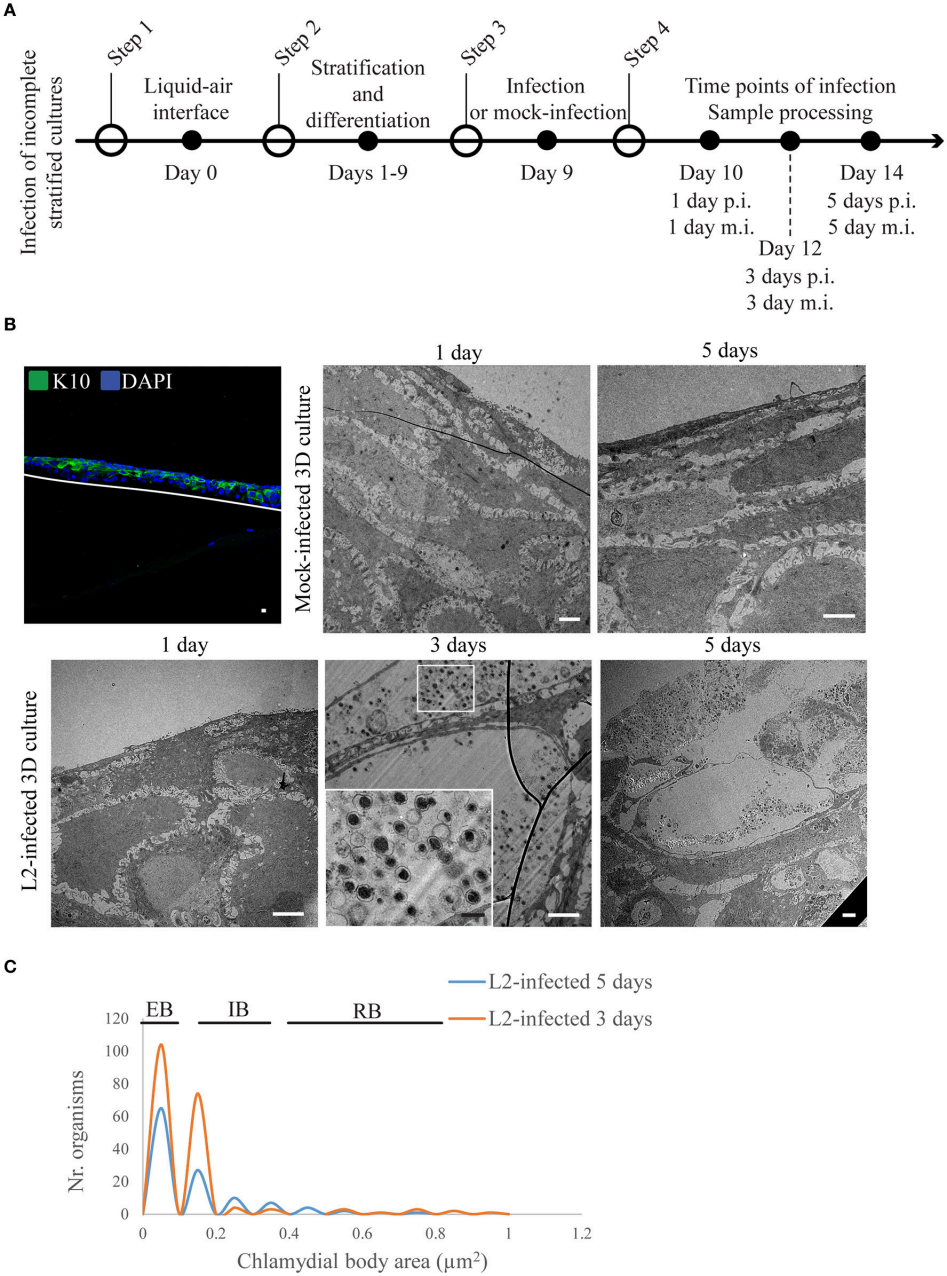
*Chlamydia trachomatis* completes its developmental cycle in early differentiated layers of the 3D organotypic cultures. **(A)** Diagram of the set up for the 3D cultures differentiation and infection. **(B)** Incomplete differentiated cultures express k10 at the top of the culture. Cultures were infected by 1, 3, or 5 days at the two latest time points *C. trachomatis* L2 inclusions containing EBS were visualized. White line represents the bottom of the 3D culture. A representative image from two independent experiments is shown. Confocal microscopy scale bar = 10 μm; TEM micrographs: white scale bar = 2 μm; black scale bar = 500 nm. **(C)** Quantification of inclusion contents and the area of EBs, RBs, intermediate bodies and enlarged RBs. The plot indicates the frequency for each particle size.

We investigated the interaction of *C. trachomatis* serovar L2 with the undifferentiated (i.e., ki67-positive layer) and differentiating layers of the 3D cultures (i.e., K10-positive layers). *In vivo*, these cells become accessible to *Chlamydia* through microabrasions that occur naturally in the stratified squamous epithelium after consensual intercourse (Norvell et al., 1984). Pathogens such as HPV can take advantage of the microabrasions in the tissue to reach more hospitable cells where they can establish infection. Therefore, in our system, we investigated if the same basal cell types are much better hosts for *C. trachomatis* than the fully differentiated cells found in the top layer. We attempted to mimic microabrations in our raft cultures by puncturing the stratified epithelia with a small gauge needle. However, we encountered two major technical difficulties. First, the depth and angle of the puncture could not be reliably reproduced because of the unevenness of the surface of the raft cultures. Second, consistently capturing the entire depth of the punctured areas during histological sectioning proved to be difficult. Therefore, we resorted to the direct inoculation of undifferentiated or differentiating layers. While we were able to perform infections of layers at a defined differentiation state, we are cognizant of an important caveat to our strategy, which is that infection was performed in the absence of the additional layers that surrounds the infection site, and thus could potentially affect its progression. Inclusion growth and *C. trachomatis* L2 developmental forms were monitored during infection of undifferentiated layers (i.e., prior to lifting at the air-liquid interface) and intermediately differentiating layers when the culture was lifted at the air-liquid interface for a defined duration prior to *C. trachomatis* L2 infection (Figures 3A, 4A). When undifferentiated cells were infected with *C. trachomatis*, and then allowed to stratify and differentiate, inclusions were present at 1 d p.i. At 3 d p.i. (and thus 3 d post-lifting to air-liquid interface) mature inclusions with clearly visible EBs and RBs (Figure 3B) were observed. *C. trachomatis* L2 developmental forms present at 1 and 3 d p.i. were characterized (Figure 3C). We acquired TEM images, and measured the area of the organisms from 1- to 3-d samples. In the two inclusions found at 1 d p.i., the area of the *C. trachomatis* L2 organisms was between 0.2 and 0.3 μm^2^, which was smaller than RBs (0.4–0.65 μm^2^) from samples shown in Figure 1D (dashed line) and Figure 3C (yellow line). The lack of condensed nucleoid indicated that these forms may have been in the middle of the EB-to-RB transition. However, the size distribution of chlamydial organisms from five inclusions at 3 d p.i. indicated the presence of EBs, IBs, and RBs, suggesting that between 1 and 3 d p.i., *C. trachomatis* L2 replicated and completed its developmental cycle. The majority of the *C. trachomatis* L2 forms had areas of 0.15 μm^2^. The areas of RBs ranged from 0.4 to 0.65 μm^2^, although a few large ones were found (0.95–1.15 μm^2^). Based on morphometric measurements, we conclude that proliferating undifferentiated HaCaT cells supported the completion of the developmental cycle, with inclusions harboring all known developmental forms.

We also investigated chlamydial growth when infecting layers in the middle of differentiation (i.e., positive for K10) by inoculating the raft cultures at day 9 post-lifting at the air-liquid interface (Figure 4A). At this time point, it was clear that there were less layers when compared to a fully stratified day 19 raft culture (Supplementary Figure 2), and the upper layers expressed K10 (Figure 4B). We focused our attention on the 3 and 5 d p.i. groups as they yielded sufficient numbers of inclusions for analysis (Figure 4B). Quantification of *C. trachomatis* L2 particle area from two independent experiments showed that in both time points (3 and 5 d p.i.) the majority of particles harbored condensed nucleoid, and had areas between 0.05 and 0.15 μm^2^ consistent with EB forms (Figure 4C). RBs were found in the 3 d p.i. samples ranging from 0.35 to 0.85 μm^2^, and one enlarged RB with 0.95 μm^2^. By 5 d p.i. the trend was skewed toward EBs with some intermediate forms with areas ranging from 0.25 to 0.35 μm^2^, and RBs ranging between 0.45 and 0.75 μm^2^ (Figure 4C), indicating ongoing development. These observations were in stark contrast to those samples where the top-most layers had inclusions with condensed nucleoid-negative forms that resembled neither EBs nor RBs with regards to size (Figure 1C).

**Figure 5:**
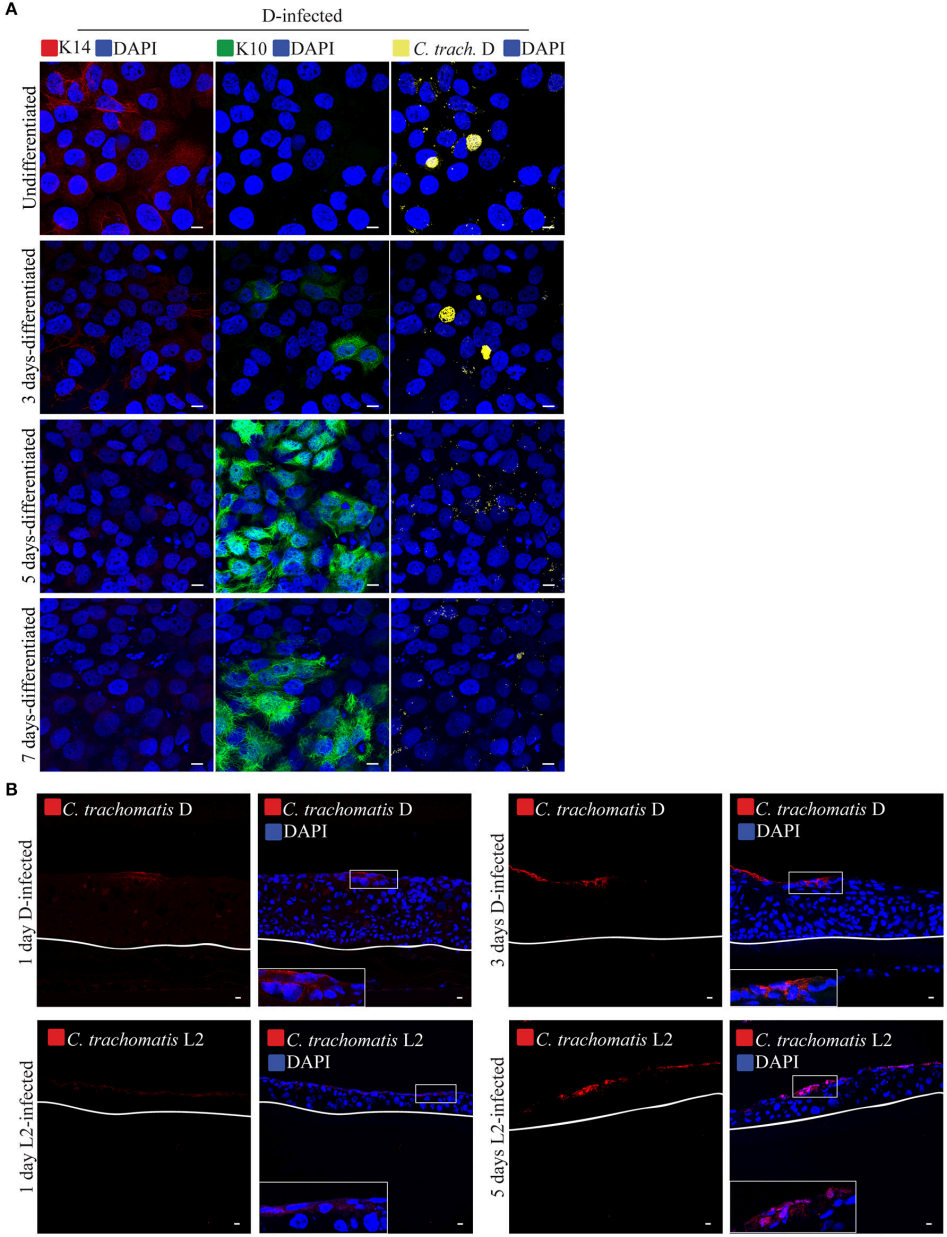
Calcium-induced differentiation of HaCaT cells decreases the infectivity of *C. trachomatis* D. **(A)** HaCaT cells in a monolayer system were exposed to high concentration (2 mM) of calcium (Ca^2+^) to induce differentiation up to 7 days. HaCaT exposed to calcium were infected with *C. trachomatis* D (without centrifugation). Infection was allowed to proceed for 24 h. Chlamydial inclusions decreased in samples exposed to high [Ca^2+^] *C. trachomatis* inclusions were stained with covalescent human sera (in yellow). A representative image from three independent experiments is shown. Scale bar = 10 μm. **(B)** *C. trachomatis* L2 or D inoculum was introduced to the top of the 3D organotypic cultures. Infections were allowed to proceed for 1, 3, or 5 days. *C. trachomatis* inclusions were stained with an anti-*C. trachomatis* LPS antibody. White line represents the bottom of the 3D culture. A representative image from two independent experiments is shown. Scale bar = 10 μm.

We also evaluated the effects of differentiation on infection by *C. trachomatis* serovar D. Being a genital strain, we wanted to determine if it exhibited some form of adaptation for better growth in differentiating cells. As shown above, serovar D, like serovar L2 was able to grow in the basal layer, but not in the terminally differentiated, filaggrin-positive layers. Here, we asked if serovar D, would grow and replicate in differentiating cells in two independent configurations. Differentiation in HaCaT monolayers or stratified raft cultures was induced by exposure for different duration to high [Ca^2+^] at or lifting at the air-liquid interface, respectively. Differentiation was followed by inoculation in Ca^2+^-free media for 1 h without centrifugation. For the raft culture, infection was performed at 9 d post-differentiation, which is well ahead of the 19 days it took to acquire a fully differentiated, e.g., filaggrin-positive, upper layers. As shown in Figure 5A, serovar D infection decreased noticeably in samples exposed to high [Ca^2+^] for 5 and 7 days (data from two independent experiments). Very few, if any inclusions were present in these samples. Infection of the intermediately differentiated raft cultures also exhibited very few infected cells and greatly diminished levels of dissemination, with *C. trachomatis* serovar D organisms only found at the upper layers (Figure 5B). We conclude that at least for serovar D, the differentiation state of the host cell determined how well this serovar grew.

### *Chlamydia trachomatis* infection at differentiating layers disseminates in a penicillin-dependent manner

**Figure 6:**
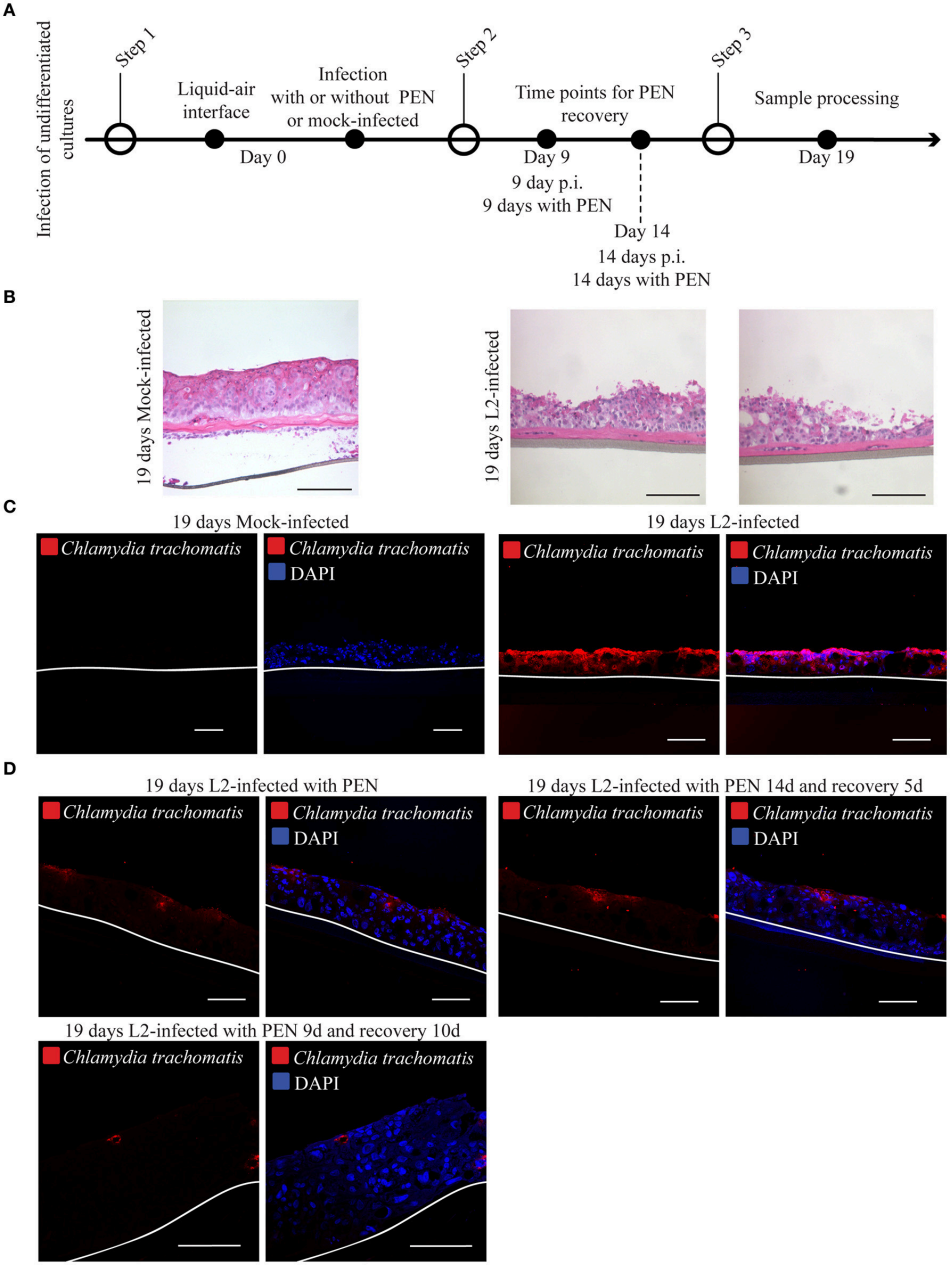
Chlamydial infection is unable to disseminate in organotypic cultures. **(A)** Diagram of the differentiation, infection and penicillin treatments. **(B)** 3D cultures were culture at liquid-air interface for 19-days in the presence (*C. trachomatis* L2-infected) or absence (mick-infected control). Samples were stain with hematoxylin and eosin. A representative image from three independent experiments is shown. Scale bar: 200 μm. **(C)** Organotypic cultures were raised to air-liquid interface to induce stratification and mock-infected (control) or *C. trachomatis* L2- infected on the same day. Cultures were grown for 19 days in the presence or absence of *C. trachomatis* L2. Samples were stained with *C. trachomatis* LPS antibody to detect infection. White line represents the bottom of the 3D culture. A representative image from three independent experiments is shown. Scale bar = 10 μm. **(D)** *C. trachomatis* L2 infections were arrested with the addition of Penicillin. Organotypic cultures were allowed to stratify and differentiate. Infection was allowed to recover from Penicillin exposure for 5 or 14 days or no recovery was allowed. No recovery or 5 days recovery showed *C. trachomatis* inclusions (red) only present on the top layers. *C. trachomatis* infection did not disseminate with the longest period of recovery, 10 days. Inclusions were detected by staining against *C. trachomatis* LPS. A representative image from two independent experiments is shown. Scale bar = 10 μm.

To gain initial insight into how infection might disseminate in stratified epithelia, HaCaT cells at 0 d post-lifting at the air-liquid interface were infected with *C. trachomatis* L2, and immediately raised to initiate differentiation. Infection was also allowed to proceed for shorter periods of time of 5 days for *C. trachomatis* L2, and 1 and 3 days for *C. trachomatis* D (Supplementary Figure 5A). At this time point it was evident that the 3D cultures were initiating the stratification process and only 1–2 cell layers have formed, and that the basal layer expressed the proliferative marker ki67 (Supplementary Figure 5B). *C. trachomatis* L2 and D infections were detectable in cryosections analyzed from four 3D cultures for *C. trachomatis* D and two independent experiments for *C. trachomatis* L2 (Supplementary Figure 5C). We focused on *C. trachomatis* L2 samples to determine if dissemination could be inhibited by penicillin. Infection was allowed to proceed for 19 days in the absence or presence of the antibiotic (Figure 6A). The samples were processed for immunostaining for *C. trachomatis* to reveal the extent of dissemination of infection. In the absence of penicillin for 19 d, infection disseminated throughout the 3D culture (Figures 6B,C), which was expected for a productively infected epithelium. Similar results were observed for *C. trachomatis* D-infected 3D cultures (Supplementary Figures 5D,E). Addition of penicillin significantly limited *C. trachomatis* L2 infection, which were confined to the upper layers. The upper layer location is likely from the continued upward displacement of cells harboring penicillin-arrested *C. trachomatis* in the stratifying raft culture.

We also included parallel cultures where penicillin was removed at either 5 or 10 d p.i. to determine if disseminated infection could be restored for the remainder of the experiment. These groups were left untreated for 14 or 9 d, respectively. As shown in Figure 6D, the extent of dissemination did not appreciably differ from the samples that were continuously exposed to the antibiotic. *Chlamydia* LPS was detected at the upper layers only. The lack of restored growth could be due to the continued presence of residual penicillin in the upper layers despite removal of penicillin.

Based on the results, *C. trachomatis* L2 appeared to exhibit preference for the undifferentiated or differentiating layers over terminally differentiated ones, while serovar D was negatively influenced by the differentiation state of the host cell. Infection of the filaggrin-positive differentiated cells in the uppermost layer was unproductive, while infection of the proliferating basal cells yielded inclusions containing EB-like organisms, indicating the completion of the developmental cycle. When allowed to proceed for 19 days, infection disseminated throughout the stratified epithelium, resulting in the significant destruction of the 3D culture. The extensive dissemination of infection was consistent with the apparent completion of the developmental cycle in the ki67-positive undifferentiated basal cell layer (Figure 3B).

## Discussion

In this study, we investigated how *C. trachomatis* interacted with three-dimensional (3D) organotypic stratified squamous models (3D cultures) to gain insight into the apparent resistance of this type of tissue to *C. trachomatis* infection as observed in clinical settings (Evans, 1982; Moorman et al., 1986). Chlamydial infection is largely studied in a monolayer system, despite the initial site encountered by *C. trachomatis* being the stratified squamous epithelium. This epithelium is characterized by multiple layers at different stages of differentiation. Cells present in the basal layer maintain the ability to proliferate, while the suprabasal cells lose this ability and commit to the differentiation program, while concomitantly displaced upwards.

Previous work by Evans et al. showed that the stratified squamous epithelium of the ectocervix of two female patients harbored very little to no chlamydial inclusions when compared to the endocervix, which consists of a single layer of columnar epithelial cells (Evans, 1982). Later, Moorman et al. observed lower levels of infection in human squamous cell cultures derived from ectocervix explants when compared to endocervical cells (Moorman et al., 1986). The authors concluded that the resistance of the ectocervical stratified squamous epithelium to chlamydial infection could be influenced by the differentiation state of these cells. In this report, we revisited this question of relative resistance of the squamous epithelium by using 3D cultures of HaCaT cells, which stratified and differentiated. A critical feature of our 3D cultures was its non-keratinizing property, along with the retention of nucleated cells at the uppermost layer. These are typical characteristics of the “wet” epithelium of the lower genital tract (Williams et al., 1988; Ryle et al., 1989; Boelsma et al., 1999; Schoop et al., 1999). We also observed desmosomes interconnecting cells within and between layers, indicating proper organization. There was some variability in thickness of the raft cultures, but their inability to support growth of *C. trachomatis* serovars L2 and D when the uppermost terminally differentiated layers were inoculated, remained consistent.

Microscopic analysis of 3D cultures infected at the top demonstrated that *C. trachomatis* inclusions remained only in the uppermost layer of the stratified epithelia despite incubations of up to 5 days. Interestingly, infection with *C. trachomatis* serovar L2 of differentiating layers, e.g., K10-positive, but filaggrin-negative yielded mature inclusions harboring all developmental forms, indicating normal progression of the developmental cycle. This is consistent with the observations made in HaCaT cells that were induced to differentiate by exposure to high [Ca^2+^] in the media for up to 7 days. These cells were fully capable of supporting the completion of the developmental cycle of serovar L2. The presence of immature inclusions among mature ones were likely due to the asynchronous nature related to inoculation without centrifugation. In contrast, we observed that *C. trachomatis* serovar D infection was inefficient in the 5- or 7-day Ca^2+^-differentiated HaCaT monolayers or in intermediately differentiating 3D raft cultures. Two independent means of differentiating cells yielded similar results, suggestive of a real effect of differentiation on the growth of serovar D. We suspect that this difference between L2 and D may be related to the known characteristic of serovar L2, which is its ability to invade into deeper tissues.

For both serovars L2 and D, infection of the upper layers failed to produce disseminated infections. We speculate that this is due to a severe delay in the developmental cycle. No inclusions containing particles consistent with EBs were found after 5 days of infection. We were not able to detect serovar D inclusions, precluding analysis of its development. While the exact cause of this developmental delay remains unknown, it may be related to the location of the host cell relative to the source of nutrients. In organotypic stratified squamous cultures, growth factors are supplied by the fibroblasts embedded within the collagen matrix, while nutrients (e.g., amino acids, co-factors, minerals, etc.) from the liquid media diffuse upwards to the epithelium, limiting nutrient access in the upper layers (Rheinwald and Green, 1975; Green et al., 1977; Lamb and Ambler, 2013). Another possibility is that nutrients are present in sufficient amounts in the upper layers, but their delivery to the inclusion does not occur. *Chlamydia* nutrient acquisition mechanisms rely on vesicular transport (Hackstadt et al., 1995, 1996; Carabeo et al., 2003; Al-Younes et al., 2004; Beatty, 2006, 2008; Robertson et al., 2009; Derré et al., 2011; Ouellette et al., 2011; Dumoux et al., 2012), which may be lost as part of the differentiation program (Sukseree et al., 2013; Li et al., 2016). An example is the presence of vesicles containing whorls that resemble autophagosomes, which have been implicated as potential sources of amino acids (Al-Younes et al., 2004; Ouellette et al., 2011), which despite their abundance, may not interact properly with the chlamydial inclusions, thus failing to deliver their amino acid content. Sphingomyelin content increases as the cells differentiate, and this lipid has been reported to be required for *C. trachomatis* differentiation (Elias et al., 1977, 1979; Lampe et al., 1983; Holleran et al., 2006; Robertson et al., 2009). It is possible that the arrested development of *C. trachomatis* in the upper layers could be related to the failed transport of sphingomyelin to the inclusions. Another potential contributing factor is iron. Previously, we reported that *C. trachomatis* acquired iron by interacting with the slow recycling pathway of transferrin receptors. Subversion of this transport pathway overcomes the lack of siderophore biosynthetic capability *in C. trachomatis* (Ouellette and Carabeo, 2010). Gatter et al. reported that in stratified squamous epithelia, transferrin receptors are only expressed by the basal cells (Gatter et al., 1983). The uppermost layers do not express transferrin receptors, and thus are incapable of providing iron to *C. trachomatis* via the slow recycling pathway.

Interestingly, we did not observe significant numbers of aberrant RBs, which are the typical hallmarks of nutritional or immunological stress. Our observations were consistent with delayed growth, rather than persistence-related abnormal development. An interesting possibility is that aberrant RBs may be relevant to infection of columnar epithelia, like those found in the endocervix, and less so to infection of the stratified squamous epithelia of the lower genital tract. Indeed, enlarged RBs were more frequently found in human endocervix samples (Lewis et al., 2014). That persistent forms were more often found in columnar epithelial cells of the upper genital tract than stratified squamous epithelia of the lower genital tract may indicate different mechanisms of adaptation in the two environments. A comparative transcriptomic analysis of the chlamydial organisms growing in either the upper layers of the stratified epithelia or simple columnar epithelia might provide deeper insight into the biology of *C. trachomatis* growing in the two distinct environments.

The interaction of *Chlamydia* with the epithelium is complex with competing adaptation mechanisms employed by both the host and the pathogen, and the use of *in vitro*-generated stratified epithelia revealed a potential anti-chlamydial strategy associated with the terminally differentiated layers. This strategy would not have been revealed in cultured cell monolayers, highlighting the value of 3D cultures. While cell cultures serve as a simple and genetically tractable model, it does not retain important features of a “natural infection” (Manire and Galasso, 1959; Moulder, 1969; Hatch, 1975). The delayed growth observed in the 3D cultures used in this study may better reflect the growth kinetics and the physiological conditions of the pathogen *in vivo*. An interesting question is why *C. trachomatis* has not adapted to deal with the suboptimal environment of the stratified epithelia of the lower genital tract, given that these are the sites first encountered during infection. The lymphogranuloma serovar L2 was able to infect and proliferate in intermediately differentiated layers, which contrasted with observations for the genital serovar D; and this may indicate adaptation. However, we believe that this is more related to the general robustness of L2 with regards to growth and replication, rather than a specific adaptation to growth in stratified epithelia, whereas serovar D, which is a slower growing strain of *C. trachomatis* may not be able to overcome growth inhibition related to the differentiation state of the HaCaT cells. There is also the possibility that serovar D may not grow efficiently in undifferentiated HaCaT cells, similar to what has been reported for serovar E (Joubert and Sturm, 2011). Affecting the differentiation state of the host cell by reversing it to a more undifferentiated state would be an interesting adaptation mechanism. If such a mechanism existed, there would be biologically interesting implications on pathology and the potential link to cervical carcinogenesis. There have been a few reports that attempted to establish a link between *C. trachomatis* infection and cervical cancer (Knowlton et al., 2013), but no molecular mechanisms have been identified to support this idea. Also, if *C. trachomatis* could modulate the differentiation state of the host, the course of infection by HPV would expectedly be affected, given that the viral life cycle and replication are intimately tied to the keratinocyte differentiation process. There have been a number of reports that describe the co-incidence of HPV infection with *C. trachomatis*. To address this, a more detailed elucidation of the differentiation process would be required to maximize detection of subtle, but biologically relevant changes to the differentiation process of the stratified epithelium.

Our combined results point to a model whereby the stratified squamous epithelium, when intact, presents a natural barrier to *C. trachomatis* infection. It offers a site for adhesion, committing *C. trachomatis* to infection. But because of the physiological state of the cells at the topmost layer, further development of the pathogen is delayed or cease altogether. However, if the barrier is breached through microabrasion, the more hospitable cell types deeper in the stratified squamous epithelium are accessed by the pathogen, and replication and dissemination ensue. If it remains unchecked, this would leads to the loss of barrier integrity increasing the chances of infections by secondary pathogens.

## Author contributions

AN designed and performed the experiments, analyzed data, and wrote the manuscript. KB provided the HaCaT cells, antibodies to differentiation markers, and suggestions in generating organotypic stratified squamous cultures. RC designed experiments, analyzed data, and wrote the manuscript.

### Conflict of interest statement

The authors declare that the research was conducted in the absence of any commercial or financial relationships that could be construed as a potential conflict of interest.

## Supplementary material

Supplementary Figure 1 Epithelial morphology of organotypic co-cultures for different fibroblasts concentration. Epithelial tissue was cultured at liquid-air interface for 14-days with murine fibroblasts. HaCaT cells formed epithelia, and their thickness was dependent on the number of fibroblasts embedded on the collagen. Hematoxylin and eosin staining; white line represents the bottom of the 3D culture. A representative image from two independent experiments is shown. Scale bar: 200 μm.

Supplementary Figure 2 Epithelial 3D organotypic co-culture goes through the differentiation process. **(A)** Cultures with 4 × 10^4^ fibroblasts embedded in collagen display basal K14 expression. **(B)** Markers of early differentiation K10. **(C)** Basal layer displays the proliferative marker ki67. **(D)** The terminal differentiation marker filaggrin is detected in the uppermost layers. White line represents the bottom of the 3D culture. A representative image from two independent experiments is shown. White scale bar: 10 μm; **(E)** 3D cultures exposed to liquid-air interface are able to form a stratified epithelium that forms desmosomes. **(F)** High magnification of the region in the inset. White arrowheads point at desmosomes. A representative image from two independent experiments is shown. Black scale bar: 1 μm.

Supplementary Figure 3 HaCaT is able to sustain *C. trachomatis* serovar L2 growth. **(A)** HeLa or HaCaT cells were infected with *C. trachomatis* L2 up to 36 h. *C. trachomatis* L2 inclusions (in red and ^*^) in HaCaT cells are similar in appearance as those in the HeLa cell control samples. *C. trachomatis* L2 inclusions were visualized with an anti-*Chlamydia* LPS antibody and DAPI stains the eukaryotic DNA and the individual bacterial DNA (small dots). A representative image from two independent experiments is shown. **(B)** Data is presented as mean of recoverable inclusion forming units (IFU), a measure for chlamydial infectious particles. IFUs were recovered at different time points of infection for both cells lines. HaCaT shows similar production of infectious particles for all time points when compared with HeLa cells.

Supplementary Figure 4 *Chlamydia trachomatis* D infection is delayed in 3D organotypic cultures. **(A)** Diagram of the 3D cultures set up for the differentiation, stratification and infection stages. **(B)** *C. trachomatis* D inclusions (red) in organotypic cultures infected for 1 d were only present on the top-most layers. By 3 d p.i. inclusions were almost absent. An anti-*C. trachomatis* LPS antibody was used to detect infection. White line represents the bottom of the 3D culture. A representative image from four 3D cultures is shown. Scale bar = 10 μm.

Supplementary Figure 5 *Chlamydia trachomatis* completes its developmental cycle in early differentiated layers of the 3D organotypic cultures. **(A)** Diagram of the set up for the 3D cultures differentiation and infection. **(B)** Undifferentiated layers express ki67 at the basal layer of the culture. A representative image from two independent experiments is shown. White line represents the bottom of the 3D culture. Scale bar: 10 μm. **(C)** 3D cultures were infected or mock-infected by 1, 3, or 5 days. *C. trachomatis* L2 and D inclusions were detected in all time points. A representative image from two independent experiments is shown. White line represents the bottom of the 3D culture. Scale bar: 10 μm. **(D)** 3D cultures infected for 19 days with *C. trachomatis* D were stained with hematoxylin and eosin A representative image from four 3D cultures is shown. Scale bar: 200 μm. **(E)** When *C. trachomatis* serovar D infection was allowed to proceed for 19 days, infection was disseminated throughout the 3D culture. Infection was detected by using an anti-*C. trachomatis* LPS antibody. A representative image from four 3D cultures is shown Scale bar = 10 μm.
