# Supplementary material for: Characterization of the Growth of Chlamydia trachomatis in In Vitro-Generated Stratified Epithelium
Source: Front Cell Infect Microbiol. 2017 Oct 10;7:438. doi: 10.3389/fcimb.2017.00438 (PMC5641298; doi:10.3389/fcimb.2017.00438)
Supplement: Supplementary Figure 1 — Epithelial morphology of organotypic co-cultures for different fibroblasts concentration. Epithelial tissue was cultured at liquid-air interface for 14-days with murine fibroblasts. HaCaT cells formed epithelia, and their thickness was dependent on the number of fibroblasts embedded on the collagen. Hematoxylin and eosin staining; white line represents the bottom of the 3D culture. A representative image from two independent experiments is shown. Scale bar: 200 μm. [file Image1.PDF]

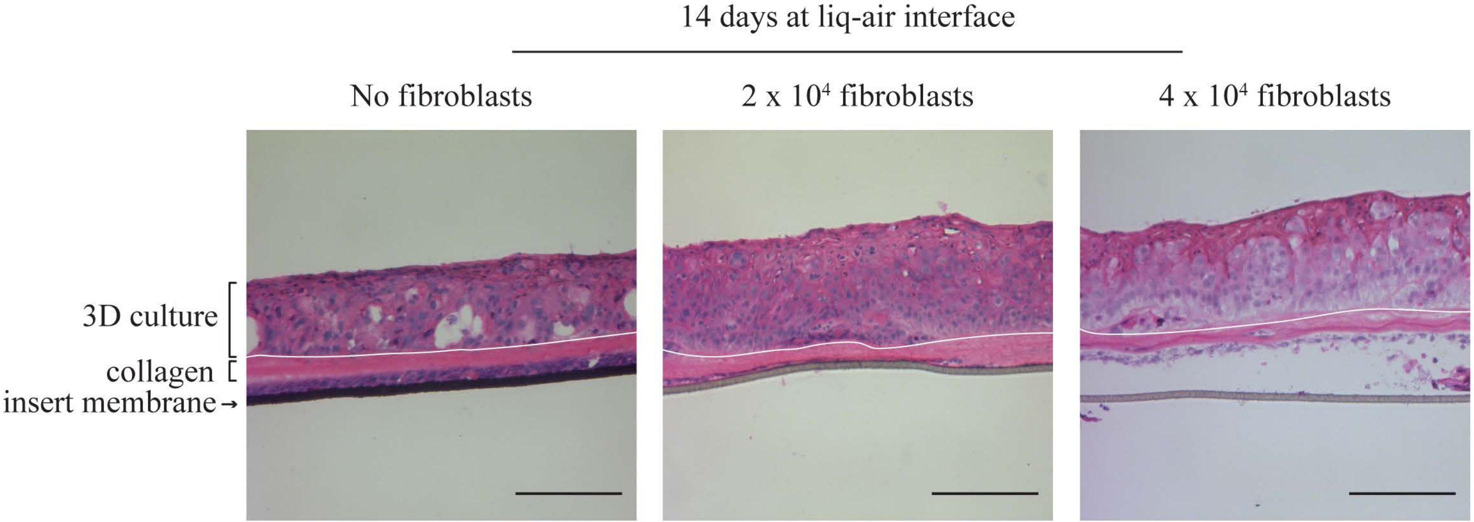
14 days at liq-air interface
No fibroblasts
2 x 10⁴ fibroblasts
4 x 10⁴ fibroblasts
3D culture
collagen
insert membrane
