# Supplementary figures and images for: Characterization of the Growth of Chlamydia trachomatis in In Vitro-Generated Stratified Epithelium
Source: Front Cell Infect Microbiol. 2017 Oct 10;7:438. doi: 10.3389/fcimb.2017.00438 (PMC5641298; doi:10.3389/fcimb.2017.00438)

A

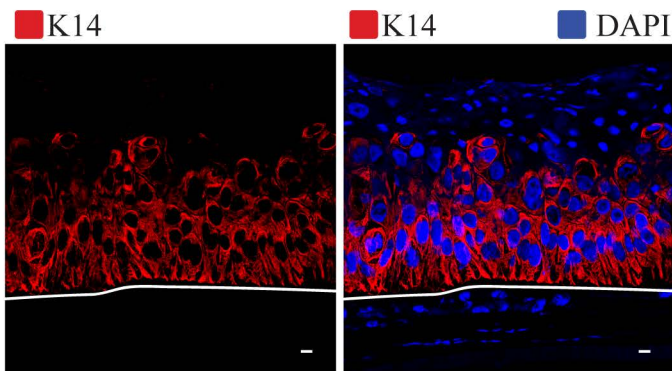

C

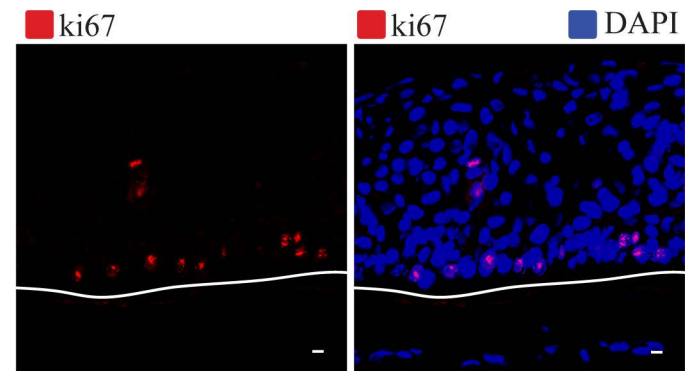

B

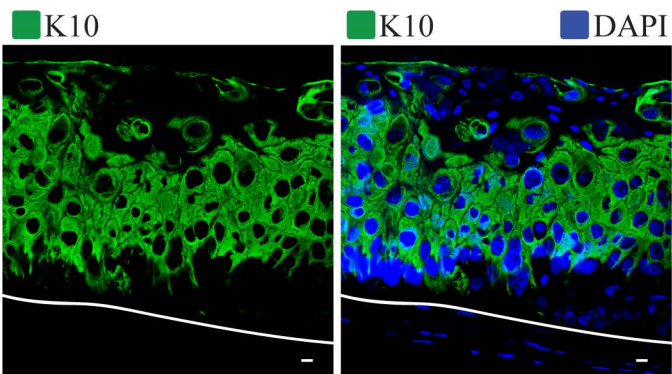

D

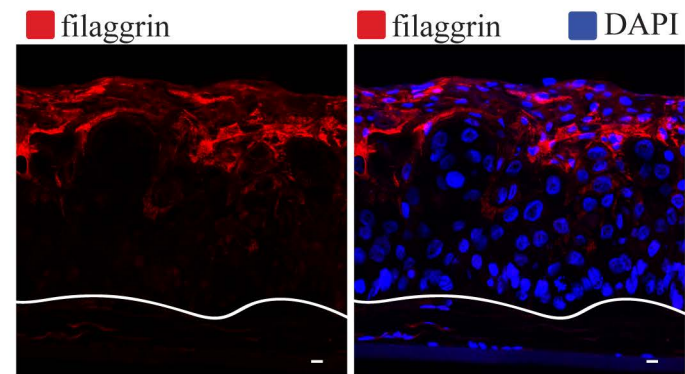

E

Mock-infected 3D culture

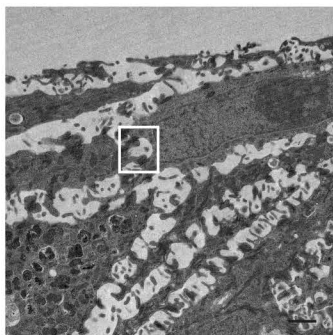

F

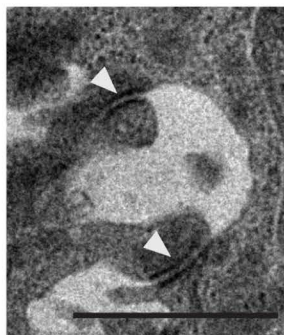

Supplement: Supplementary Figure 2 — Epithelial 3D organotypic co-culture goes through the differentiation process. (A) Cultures with 4 × 10^4^ fibroblasts embedded in collagen display basal K14 expression. (B) Markers of early differentiation K10. (C) Basal layer displays the proliferative marker ki67. (D) The terminal differentiation marker filaggrin is detected in the uppermost layers. White line represents the bottom of the 3D culture. A representative image from two independent experiments is shown. White scale bar: 10 μm; (E) 3D cultures exposed to liquid-air interface are able to form a stratified epithelium that forms desmosomes. (F) High magnification of the region in the inset. White arrowheads point at desmosomes. A representative image from two independent experiments is shown. Black scale bar: 1 μm. [file Image2.PDF]

A

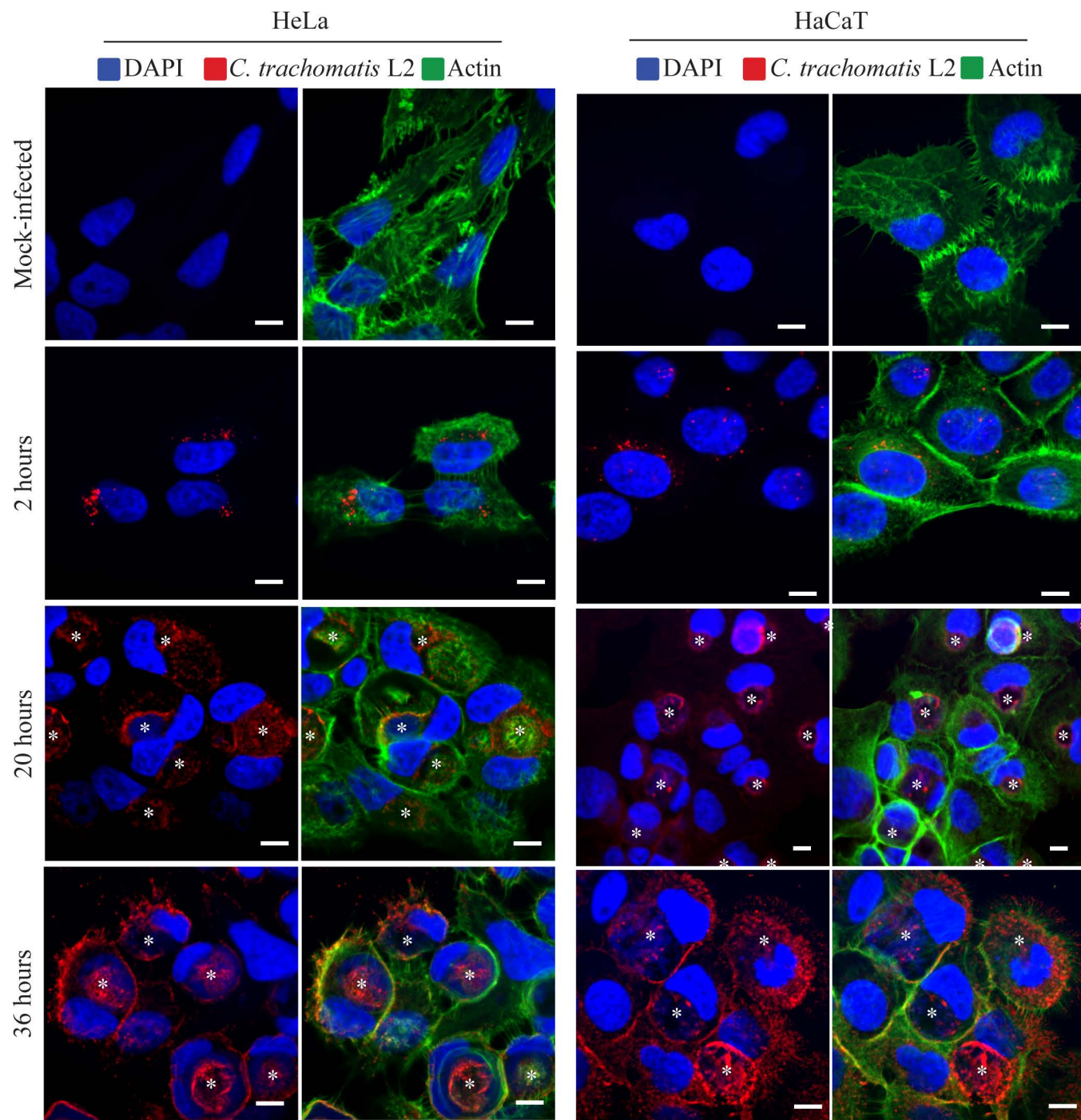

B

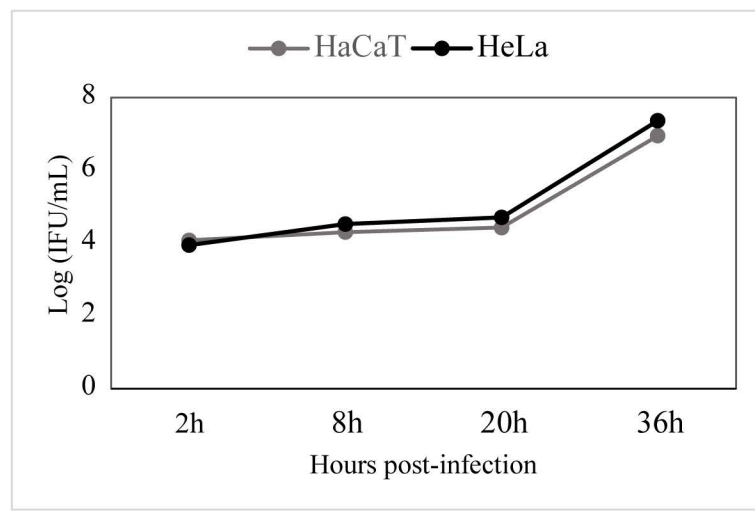

Supplement: Supplementary Figure 3 — HaCaT is able to sustain C. trachomatis serovar L2 growth. (A) HeLa or HaCaT cells were infected with C. trachomatis L2 up to 36 h. C. trachomatis L2 inclusions (in red and *) in HaCaT cells are similar in appearance as those in the HeLa cell control samples. C. trachomatis L2 inclusions were visualized with an anti-Chlamydia LPS antibody and DAPI stains the eukaryotic DNA and the individual bacterial DNA (small dots). A representative image from two independent experiments is shown. (B) Data is presented as mean of recoverable inclusion forming units (IFU), a measure for chlamydial infectious particles. IFUs were recovered at different time points of infection for both cells lines. HaCaT shows similar production of infectious particles for all time points when compared with HeLa cells. [file Image3.PDF]

A

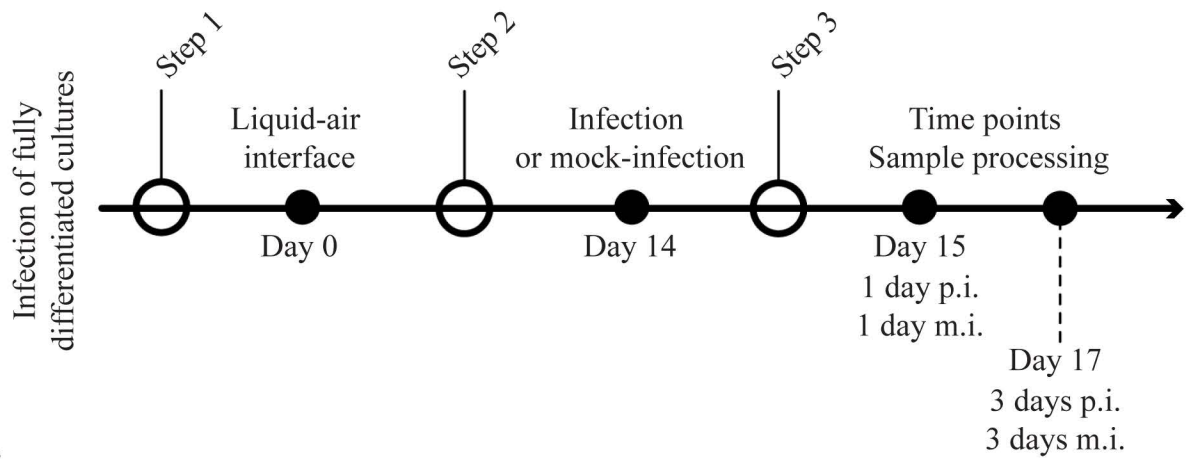

B

Mock-infected 1 day

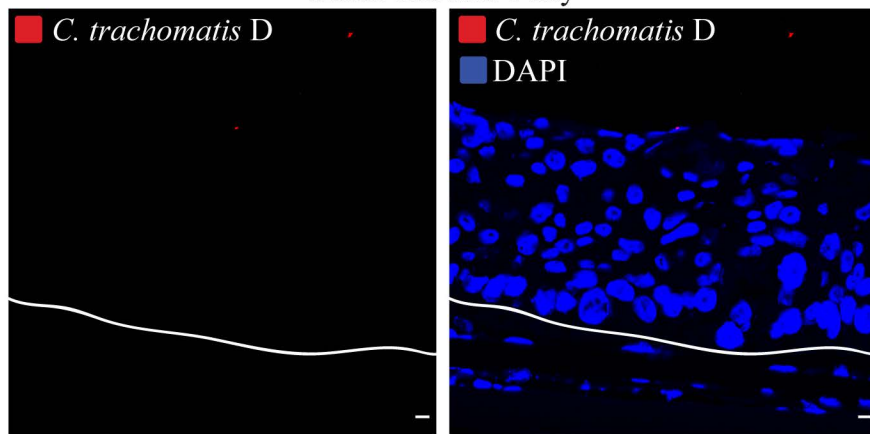

D-infected 1 day

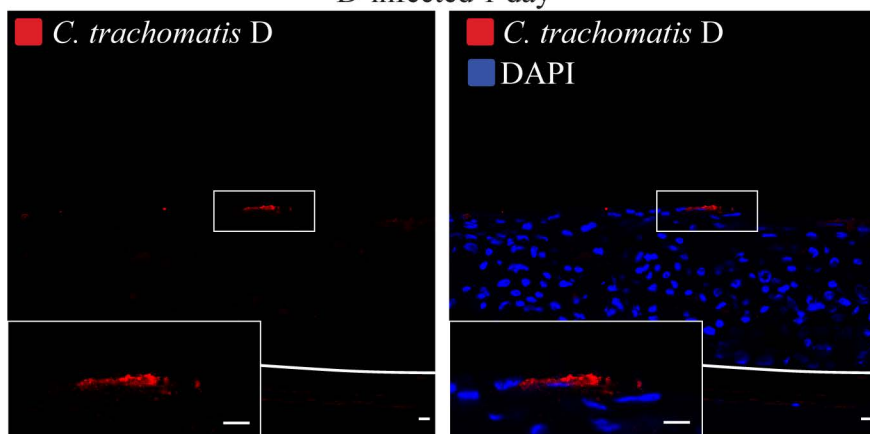

D-infected 3 days

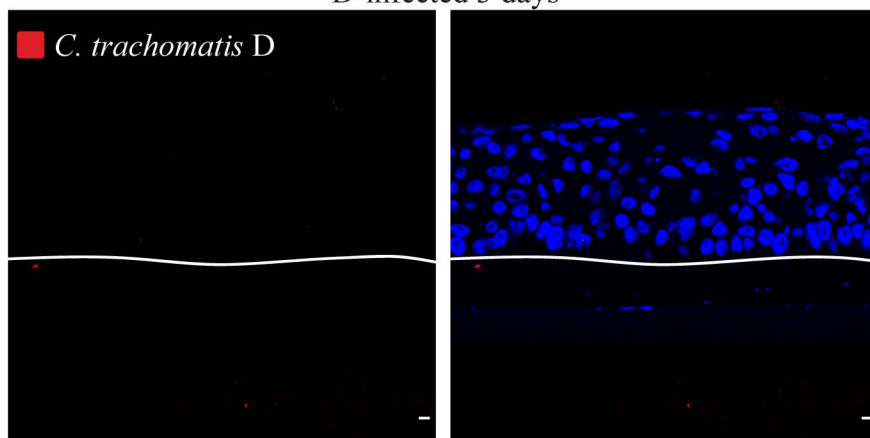

Supplement: Supplementary Figure 4 — Chlamydia trachomatis D infection is delayed in 3D organotypic cultures. (A) Diagram of the 3D cultures set up for the differentiation, stratification and infection stages. (B) C. trachomatis D inclusions (red) in organotypic cultures infected for 1 d were only present on the top-most layers. By 3 d p.i. inclusions were almost absent. An anti-C. trachomatis LPS antibody was used to detect infection. White line represents the bottom of the 3D culture. A representative image from four 3D cultures is shown. Scale bar = 10 μm. [file Image4.PDF]

A

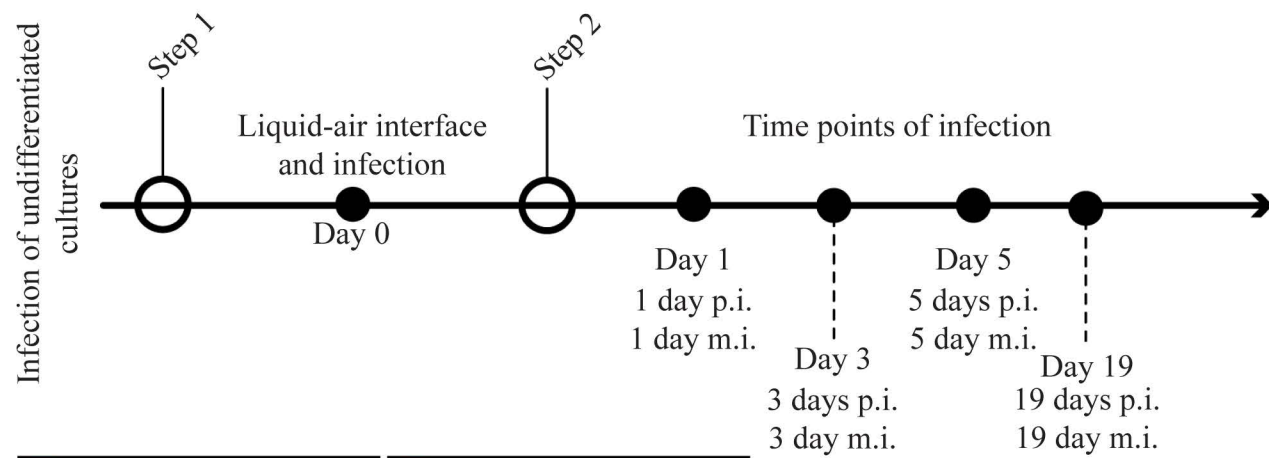

B

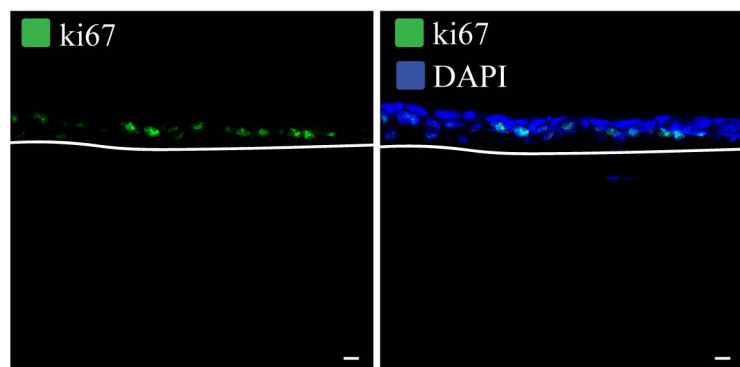

C

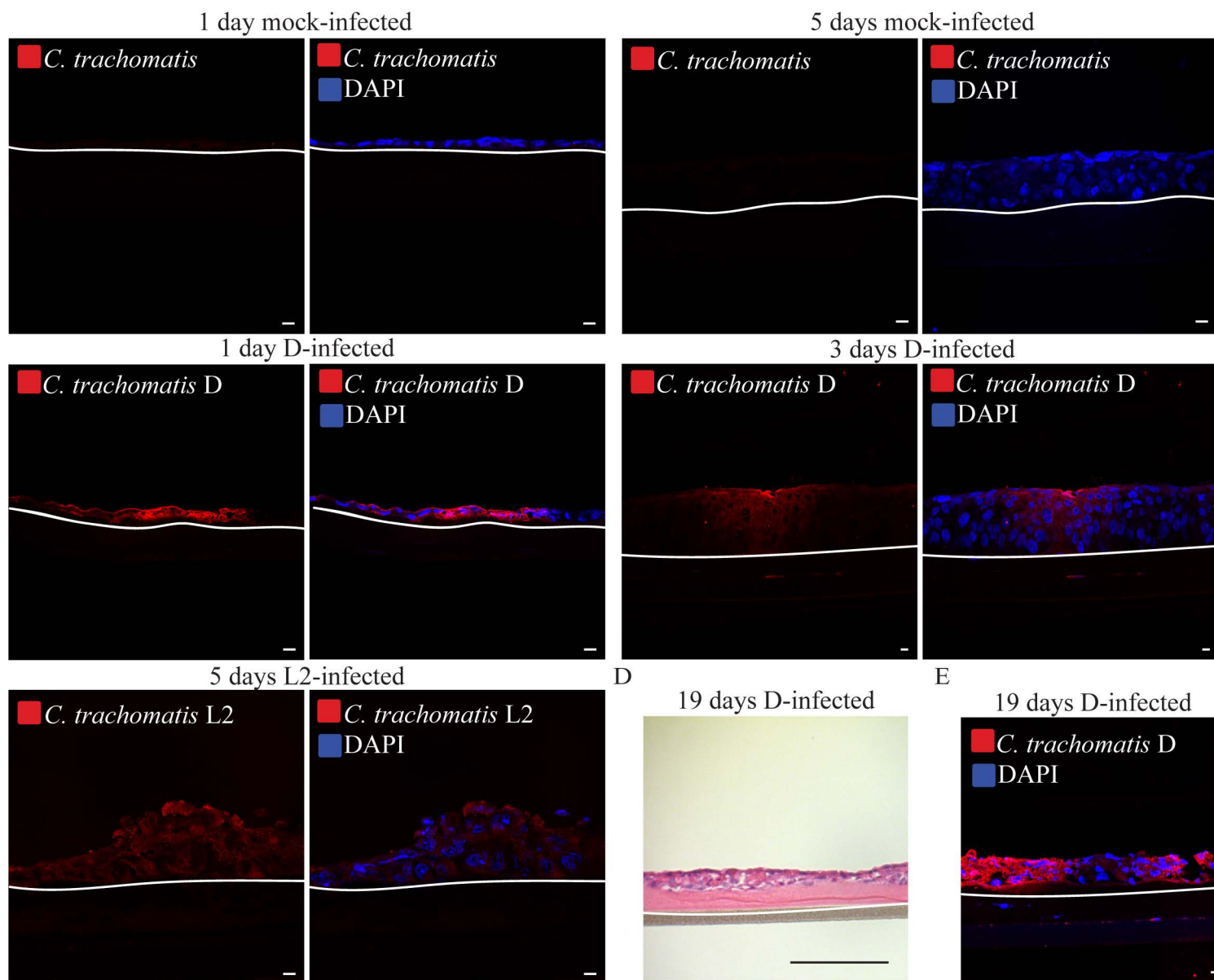

Supplement: Supplementary Figure 5 — Chlamydia trachomatis completes its developmental cycle in early differentiated layers of the 3D organotypic cultures. (A) Diagram of the set up for the 3D cultures differentiation and infection. (B) Undifferentiated layers express ki67 at the basal layer of the culture. A representative image from two independent experiments is shown. White line represents the bottom of the 3D culture. Scale bar: 10 μm. (C) 3D cultures were infected or mock-infected by 1, 3, or 5 days. C. trachomatis L2 and D inclusions were detected in all time points. A representative image from two independent experiments is shown. White line represents the bottom of the 3D culture. Scale bar: 10 μm. (D) 3D cultures infected for 19 days with C. trachomatis D were stained with hematoxylin and eosin A representative image from four 3D cultures is shown. Scale bar: 200 μm. (E) When C. trachomatis serovar D infection was allowed to proceed for 19 days, infection was disseminated throughout the 3D culture. Infection was detected by using an anti-C. trachomatis LPS antibody. A representative image from four 3D cultures is shown Scale bar = 10 μm. [file Image5.PDF]
